# Identification of circRNAs in the Liver of Whitespotted Bamboo Shark (*Chiloscyllium plagiosum*)

**DOI:** 10.3389/fgene.2020.596308

**Published:** 2020-12-11

**Authors:** Wenjie Zhang, Ping Qin, Xiaoxia Gong, Lei Huang, Chan Wang, Guiqian Chen, Jianqing Chen, Lei Wang, Zhengbing Lv

**Affiliations:** ^1^College of Life Sciences, Zhejiang Sci-Tech University, Hangzhou, China; ^2^Zhejiang Provincial Key Laboratory of Silkworm Bioreactor and Biomedicine, Zhejiang Sci-Tech University, Hangzhou, China; ^3^Hangzhou Hongqiao Sino-Science Gene Technology Co., Ltd., Hangzhou, China

**Keywords:** circRNA, back-splicing, *Chiloscyllium plagiosum*, miRNA, liver homeostasis

## Abstract

Whitespotted bamboo shark (*Chiloscyllium plagiosum*), a member of the cartilaginous fish family, has an extremely large liver and demonstrates a strong regeneration ability and immune regulation. Circular RNAs (circRNAs) is an important class of non-coding RNAs. Increasing evidences suggest that circRNAs are a kind of potential regulators. Recently, researchers have isolated and identified different circRNAs from various species, while few reports were on the circRNAs of *C. plagiosum*. In this study, we have identified a total of 4,558 circRNAs in the liver of *C. plagiosum*. This finding suggests that circRNAs are not evenly distributed in the chromosomes and follow the GT-AG rule during cyclization. Alternative back-splicing might exist in shark circRNAs as shown by the authenticity identification of predicted circRNAs. The binding strength of circRNAs (<2,000 bp) and the detected miRNAs in shark liver were simultaneously analyzed to construct an mRNA–miRNA–circRNA network for the Glutathione S-transferase P1 gene, and the circRNA authenticity was simultaneously verified. Our data provide not only novel insights into the rich existence of circRNAs in marine animals, but also a basis for characterizing functions of identified circRNAs in the liver homeostasis of *C. plagiosum*.

## Introduction

Approximately 530 million years ago, cartilaginous fishes diverged from jawed vertebrates, which are the common ancestors of teleost fish and humans. Similar to teleost fishes, cartilaginous fishes have complex physiological systems that include an adaptive immune system and a pressurized circulatory system. It is the first jawed vertebrate with an adaptive immune system. A member of the cartilaginous fish family, whitespotted bamboo shark (*Chiloscyllium plagiosum*), is mainly distributed in the Indo-West Pacific waters around several East Asian countries, such as Singapore and Indonesia. This marine animal has important research and commercial food value. In contrast to other animals, *C. plagiosum* has a large liver, accounting for approximately 75% of the total weight of its internal organs. The liver is not only an important organ with detoxification function but also has a strong regeneration ability, which endows *C. plagiosum* with many special characteristics, such as strong immune regulation (Zhou et al., 2011; Wang et al., 2013; Zhang et al., 2013; Masstor et al., 2014).

CircRNAs, a novel type of non-coding RNAs (ncRNAs) (Jeck and Sharpless, 2014; Hsiao et al., 2017), form covalent-closed continuous loops without 5′ to 3′ polarities and poly(A) tails (Liang and Wilusz, 2014; Yaylak et al., 2019). Large amounts of circRNAs have been successfully identified in a variety of organisms, including plants, animals, and humans (Rybak-Wolf et al., 2015; Maass et al., 2017; Ye et al., 2017). According to the circBase statistics^1^, approximately 410,000 circRNAs of six species had been identified; however, only one type of marine animal has been detected with circRNA (Pallavicini et al., 2013b). CircRNAs, which are an important part of ncRNA families, are ubiquitously expressed in eukaryotic cells during post-transcriptional processes (Salzman et al., 2012, 2013). CircRNAs form via non-canonical splicing termed back-splicing, while the canonical splicing machinery could control pre-mRNA back-splicing (Kulcheski et al., 2016); as such, the efficiency of circularization may rely on the presence of canonical splice sites (Chen et al., 2016). CircRNAs were thought to have two unique features: high stability due to their resistance to the cellular linear RNA decay machineries, and particular structure because the circular feature may endow circRNAs with unique structure (Liu et al., 2019). Increasing evidence suggests that circRNAs are a kind of potential RNA regulators, and their abnormal expressions are correlated with various human diseases, especially cancer in humans (Meng et al., 2018).

CircRNAs have multiple biological functions: they serve as miRNA sponges or bind to essential proteins as RNA-binding proteins, regulate alternative splicing and gene expression, act as templates for translation, and play other unknown roles (Hansen et al., 2013; Ashwal-Fluss et al., 2014; Li et al., 2015). Emerging evidence suggests that circRNAs can collectively bind and suppress the activation of the kinase PKR, thereby controlling innate immune response (Liu et al., 2019), which is the first line of defense against invading pathogens (Mogensen, 2009). In addition, circRNAs are involved in antiviral immunity; the circRNA–miRNA–mRNA network could regulate host immune function (Chen et al., 2017; He et al., 2017; Zhang et al., 2017), and thus provide efficient protection against viral infections (Wang et al., 2017b). Certain identified circRNAs could activate retinoic acid-inducible gene-I (RIG-I) and confer effective immune protection against viral infections (Chen et al., 2017). Glutathione S-transferases (GSTs) are multifunctional enzymes that are primarily involved in cellular defense against toxins in most living organisms (Hayes et al., 2004). Glutathione S-transferase kappa 1 (GSTκ1) from the big belly seahorse (*Hippocampus abdominalis*) represents an important role in innate immunity and detoxification of harmful xenobiotics (Samaraweera et al., 2019). The GST kappa from *Haliotis discus* responds against immune and stress challenges (Sandamalika et al., 2018). Glutathione S-transferase P1 (GSTP1) is a member of the GST enzyme superfamily and two miRNAs have been verified to target the GSTP1 3′UTR region in the liver of *C. plagiosum* (Ge et al., 2017).

In this study, circRNAs in the liver of *C. plagiosum* were identified, and their binding strength (<2,000 bp) was determined; the detected miRNAs in shark liver were simultaneously analyzed to construct an mRNA–miRNA–circRNA network for the GSTP1 gene; two of indentified circRNAs that are predicted to interact with each other were cloned on the dual luciferase vector PsicHECK 2. The miRNA was co-transfected into 293T cells, and finally tested by dual luciferase to verify the interaction between circular RNA and miRNA, as well as the authenticity of circRNA to expand current understanding on circRNAs in marine animals (Meng et al., 2018; Zhao et al., 2020).

## Materials and Methods

### Animals

An adult male *C. plagiosum* with length of 40 cm was obtained from the East China Sea. The shark was anesthetized using MS-222, and its liver was collected and stored in liquid nitrogen (Ohta et al., 2000). All procedures were approved by the Zhejiang Sci-tech University Animal Experimental Ethics Committee.

### CircRNA High-Throughput Sequencing

In brief, total RNA was pretreated to enrich circRNAs by using a CircRNA Enrichment kit (Cloud-seq, Inc., United States). RNA libraries were constructed using pretreated RNAs with a NEBNext^®^ Ultra II^TM^ Directional RNA Library Prep kit (New England Biolabs, Inc., Massachusetts, United States) in accordance with the manufacturer’s instructions. Libraries were controlled for quality and quantified using a BioAnalyzer 2100 system (Agilent Technologies, Inc., United States). Library sequencing was performed on an Illumina Hiseq 4500 instrument with 150 bp paired end reads.

### Divergent and Convergent Primer Design

**FIGURE 1:**
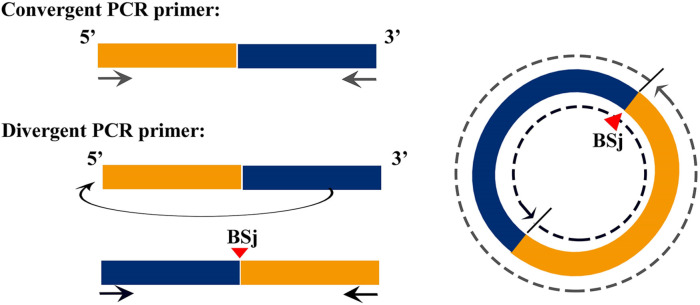
Schematic of the design of divergent and convergent primers, demonstration of the principle of forward and reverse primer design.

The correction of circRNAs identified from *C. plagiosum* were validated by randomly selecting several circRNAs from a circRNA high-throughput database. As shown in Figure 1, a PCR amplification template was made by joining a half sequence of circRNAs from the 3′ end to the 5′ end of the circRNAs. The new amplification template sequence was used to design divergent PCR primers, the initial sequence was used to design convergent PCR primers (Panda and Gorospe, 2018).

### Total RNA Isolation and Linear RNA Degradation

The total RNA of the wild whitespotted bamboo shark was extracted from the liver by using a Trizol reagent (Invitrogen, CA, United States) following the manufacturer’s procedure. An RNase R digestion reaction was prepared, the mixtures were incubated at 37°C for 30 min, and RNA was immediately isolated.

### cDNA Cloning of CircRNAs

Liver cDNA was synthesized using a PrimeScript^TM^ 1st Strand cDNA synthesis kit (Takara, Japan) in accordance with the manufacturer’s instructions, added 1 μg total RNA per 10 μL system, and used as templates for PCR amplification. The primers for RT-PCR and expected sizes are shown in Table 1. PCR was carried out in accordance with the normal regulation rules, with 2 μL cDNA added to the 50 μL system. The PCR product was separated with 2% agarose gel electrophoresis. The purified PCR product of the junction site and circular sequence of each circRNAs was further validated via Sanger sequencing.

**TABLE 1:** Sequence of RT-PCR primers.

CircRNA Name	Divergent PCR primers	Convergent PCR primers
328–177	F: TGCAAACCTGGCTTTAAACCAGT	F: TTACTTGTACAGTAAACAGGTATTTAAACAT
	R: ATTGTAAGTGATTACATCATCCACGTG	R: CAACTTGACAGGCAGGTTCTG
294–177	F: TGCGATCGTAGGTTTAAACCAGT	F: TTACTTGTACAGTAAACAGGTATTTAAAAAAT
	R: ACTGTAAGTGATTTCATCATCCATGTG	R: CAATTACACATGCAGGTTCTGGAT
50–170	F: GGTTCTAGAGCAGGAAAAAACATTGT	F: GTGTGCTGGAACACTTGAAGACAC
	R: TGCGCTTTGTGCTGTGCTTC	R: TCCAGCAGACTGTGCAGTCACT
34–434	F: GTTGCTTACCTCGCAACTACAAAAT	F: AACTATCCACCACATTCAGTTTCAC
	R: ATAATTGTTATAAACTAGCTGATTACTACTTTC	R: AGATGCCAAAGCTTTTCCAAG
30–219	F: GAGTCCACAGAACGGTTGGAT	F: AATGAAAAATGCTGGAAACAGAC
	R: ACTGAAGACATTGTTCCGGACAC	R: CTGTGGTTAAAAAGCACTTTGTG
28–237	F: CGGTTGGACGCGATTCCG	F: GTCCGCCACAGATTTCTTCAAG
	R: CTGTGAATGGTGCGGCCAT	R: TAATCACAATGAGAGTGTAGTTGAGGT
22–545	F: GGCTGTCATTGCCTTGATTTTTC	F: TGTAAGCACACCCAGTCTGGC
	R: TTGTGGGCAGAATTGATTTTAG	R: TGGCAGGTGAAGGGGTACTG
21–352	F: CACTGCAGGCATTCCACATC	F: GTTCACAATGTCTGGAAATAAACGT
	R: TGCCTTTAAATGTCGGGGC	R: CTGTGTGTTGGTGCAGGCT
38–1717	F: CAGTTTACTCCTCCGTATTCCT	F: GGTGGAGGATTGGAAGGCT
	R: AAGCCTTCCAATCCTCCAC	R: CGGGTAGGAATACGGAGGAG
6–1096	F: GTATCCTGGCACGATTAAGC	F: TGAACGGACGGAACCTTG
	R: CATCACCATTCGCACTGTTCT	R: CTGCTGGAGGATTGGCCT

**F, forward; R, reverse.**

### CircRNA–miRNA–mRNA Regulation Network Construction

TargetScan (5.0), and miRanda (3.3a) were used to predict targeting relationships (Hu et al., 2019) between circRNAs with a sequence of less than 2,000 bp and miRNA (Cheng et al., 2017). Based on the results, selected the circRNAs–miRNA [miRNAs that can combine with GSTP1 3′ UTR region (Ge et al., 2017)] pairs with the minimum free energy (mfe) <-20 kcal/mol and TargetScan_score ≥90 to construct a circRNA–miRNA–mRNA regulation network using Cytoskope software. The obtained circRNAs were identified by treating 20 μg of total RNA with 1 μL RNase R (Lucigen, United States), the RNAs treated with RNase R was used for RT-PCR. The primers are shown in Table 1.

### miRNA-mRNA-circRNA Interaction Study

Through double enzyme digestion, circRNA (38-1717 and 6-1096) predicted to interact with the dual luciferase carrier psiCHECK-2 were connected to form recombinant vectors psiCHECK-2-circ-38-1717 and psiCHECK-2-circ-6-1096, and then the interacting miRNA (ipu-miR-143 targets 38-1717, direct -7a target 6-1096) is sent to Youkang Biological Company to synthesize miRNA mimics, and then the recombinant vector and miRNA mimics are co-transfected into 293T cells.

The plasmid was extracted using Endofee Plasmid Mini Kit II (Omega, United States), 293T cells were obtained from our laboratory. Cells were cultured in DMEM (with L-Glutamine, Gibco, United States) supplemented with 10% heat-inactivated fetal bovine serum (FBS) (Gibco, United States) and 1% penicillin-streptomycin (Gibco), in an incubator containing 5% CO_2_ at 37°C In the cultivation. Twenty four–Forty eight hours prior to transfection, spread 2 × 10^5^ cells/well 293T cells in a 12-well plate and culture overnight to make the cell density about 80% during transfection.

With Lipofectamine 2000 Transfection Reagent (Thermo Fisher Scientific), the four experimental groups psiCHECK 2 + microRNA mimics-ipu-miR-143 empty plasmid control group, psiCHECK 2-38-1717 + microRNA mimics-ipu-miR-143 experimental group; psiCHECK 2 + microRNA mimics-dre-let-7a empty plasmid control group and psiCHECK 2-6-1096 + microRNA mimics-dre-let-7a experimental group were co-transfected into 293T cells. Add 1.25 μg plasmid and 4 μL liposomes, 1 μL microRNA mimics to each well. Culture cells for 48–72 h at 37°C, followed by the dual luciferase assay. Incubate the cells at 37°C for 48–72 h, and then use Dual Luciferase Reporter Gene Assay Kit (Yeasen, China) for dual luciferase assay.

## Results

### Identification of CircRNAs

The total RNAs from the liver of *C. plagiosum* were isolated to investigate circRNAs. The total RNA was treated with a circRNA Enrichment kit to enrich circRNAs and to construct libraries for deep sequencing through the Illumina HighSeq 4000 platform. Sequencing generated 64.12 million nucleotide raw base data. After adaptors were trimmed and low-quality reads were filtered, 64.03 million clean reads were obtained (Table 2).

**TABLE 2:** Reads statistics.

Sample	Raw reads	Q30	Clean reads	Clean ratio	Mapped reads	circRNA number
Wt-liver	64,124,640	92.53%	64,033,850	99.86%	42,355,809	4558

**FIGURE 2:**
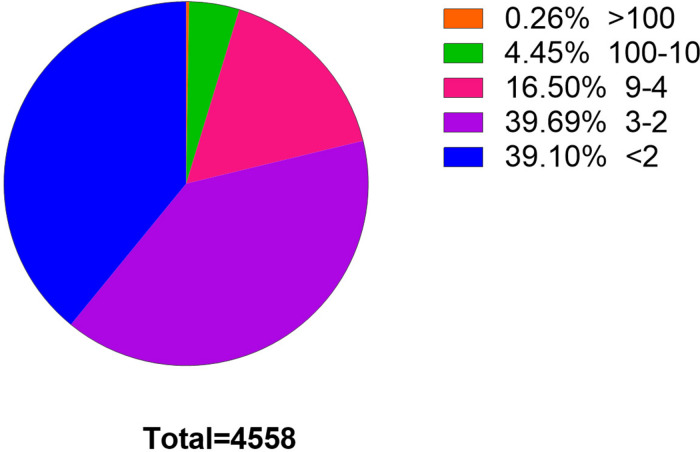
Statistics of circRNAs BSj reads. Among these 4,558 genes, only 0.26% has a reading of more than 100 BSj; 4.45% has a reading of 10–100 BSj reads; 16.5% has a reading of 4–9 BSj reads; while 39.69% for 2–3 BSj reands and 39.10% less than 2 BSj reads.

Clean reads were aligned with the *C. plagiosum* genome (ASM401019v1) by using bowtie2 software (Langmead and Salzberg, 2012), and circRNAs were detected and identified via find_circ software (Memczak et al., 2013). The principle of find_circ: basis on the result of Bowtie2 alignment, find_circ extracts 20nt anchor sequences from both ends of the reads that are not aligned to the reference sequence, and aligns each pair of anchor sequences with the reference sequence again. If the 5′end of the anchor sequence is aligned to the reference sequence (the start and stop positions are marked as A3 and A4, respectively), the 3′end of the anchor sequence is aligned to the upstream of this position (the start and stop positions are marked, respectively as A1, A2), and there is a splice site (GT-AG) between A2 and A3 of the reference sequence, then this read is used as a candidate circRNA. Finally, candidate circRNAs with read counts greater than or equal to 2 were used as identified circRNAs. The total number of matched reads was used to normalize the reverse splicing reads (junction reads) of each sample and log2 conversion. According to find_circ analysis, at least 4,558 circRNAs that had one back-splicing junction reads (BSj reads) were obtained. Among them, 2,776 circRNAs (39.1%) had more than one BSj reads, and 967 circRNAs had more than four BSj reads, accounting for 20% of the total reads (Figure 2).

### CircRNA Signature Analysis

**FIGURE 3:**
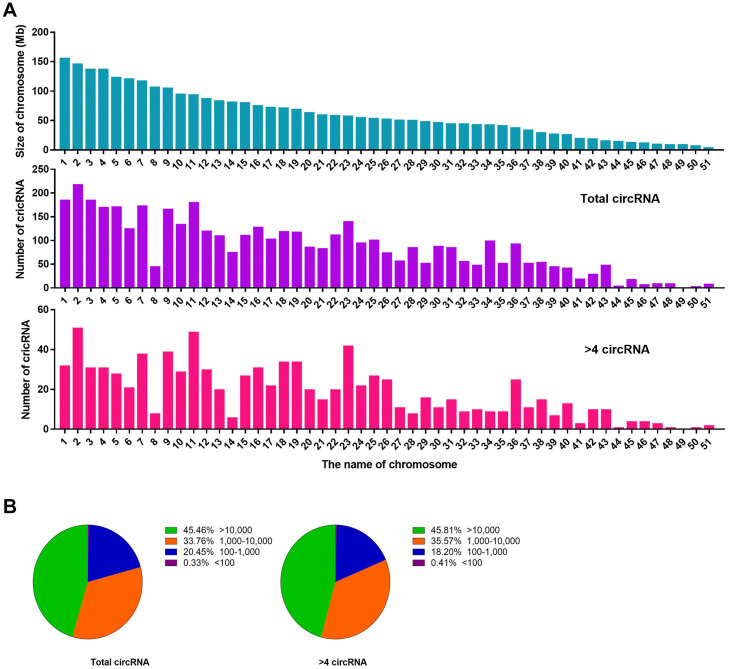
Statistics of circRNAs distribution and length. **(A)** Size of chromosomes in *C. plagiosum* (blue column) and the number of circRNAs that matched on the chromosomes (purple column represents total circRNAs; pink column represents BSj reads >4 circRNAs). **(B)** Statistics of the length of total circRNAs and BSj reads >4 circRNAs; The first pie chart shows the proportion of circular RNA from 100 to 10,000 bp in all circular RNAs; The second pie chart shows the distribution of circular RNA length from 100 to 10,000 bp in all circular RNAs with BSj greater than 4.

We performed a set of counting calculations for total circRNAs and BSj reads >4 circRNAs to determine the characteristics of liver circRNAs in *C. plagiosum*. According to NCBI database, *C. plagiosum* had a total of 51 chromosomes, ranging in size from 4.64 to 156.6 Mb. When referring to the relative expression levels using the ratio of circRNA number to genome size (Mb), the relative expression of *C. plagiosum* circRNAs (∼1.47) is slightly higher than those of humans (∼1.00). Larger number of the chromosome usually have smaller size (Figure 3A). Overall, no matter total circRNAs or BSj reads >4 circRNAs, the number of them represent a downward trend with the size of chromosome 1 to 51, and circRNAs detected on chromosome 8 are relatively smaller than those of other chromosomes of similar size. The diversity in the number of matched circRNAs on chromosomes of similar size is pronounced in BSj reads of >4 circRNAs; for example, relatively more BSj reads >4 circRNAs matched on chromosomes 2, 11, 23, and 36 than on chromosomes 8 and 14, which indicates that circRNAs are not evenly distributed on *C. plagiosum* chromosomes.

The length of the circRNAs, total circRNAs, and BSj reads >4 circRNAs exhibited a similar assignment in terms of molecular length (Figure 3B). Nearly half of the circRNAs are with a length more than 10,000 bp, which might be due to incomplete genome annotation or the original long circRNAs of sharks.

### Identification of CircRNAs

**FIGURE 4:**
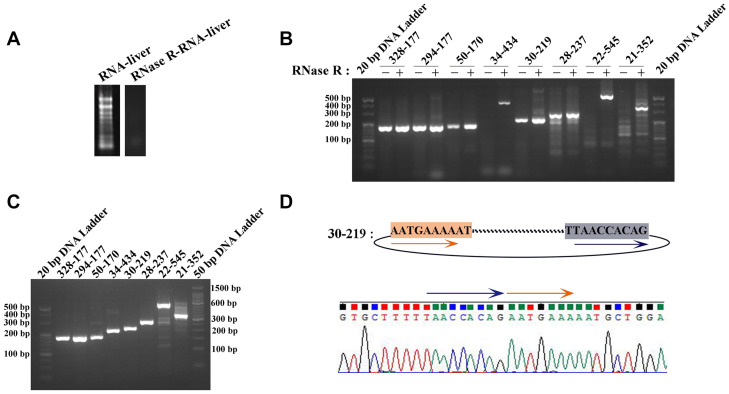
Identification of circRNAs. **(A)** The total RNA was treated by RNase R. **(B)** The amplification result of RT-PCR using divergent PCR primers (“ + ” represents RNase R-treated RNA as the reverse transcription template, “−” represents the total RNA transcription). **(C)** Amplification result of RT-PCR using convergent PCR primers and RNase R-treated RNA as the reverse transcription template. **(D)** Sanger sequencing of 30-219 confirmed the back-splicing junction in PCR products.

Two pairs of primers for RT-PCR (divergent and convergent primers) were designed to verify the authenticity of identified circRNAs from the *C. plagiosum* liver. Eight highly expressed circRNAs in *C. plagiosum* were selected (Table 3) and named “the number of BSj reads—predicted sequence length.” The back-splicing junction was detected by RT-PCR. After treated with 1 μL Ribonuclease R per 20 μg of total RNA, the results were compared before and after RNase R degradation of total RNA (Figure 4A).

**TABLE 3:** Information on eight circRNAs with high BSj reads.

CricRNA ID	Number of BSj reads	Predicted sequence length	circRNA name
QPFF01524526.1:668-844 +	328	177	328-177
CM012981.1:19191743-19191919-	294	177	294-177
CM012964.1:995687-995856 +	50	170	50-170
CM012991.1:19134334-19134767-	34	434	34-434
CM012986.1:38607300-38607518 +	30	219	30-219
CM012956.1:24938513-24938785 +	28	273	28-237
CM012988.1:10693863-10694407-	22	545	22-545
CM012971.1:29840270-29840621-	21	352	21-352

All the sequences of expected sizes of selected circRNAs have been successfully amplified (Figure 4B). At the same time, circRNAs, especially 34–434, 22–545, and 21–352, could be enriched through total RNA treated with RNase R, whereas no band was amplified when total RNA and RNase R-treated RNA were used. An RT-PCR product with an expected length could be amplified using convergent PCR primers except 34–434 (Figure 4C). 34–434 authenticity was verified by amplification using a new convergent PCR primers (Supplementary Figure 1C). CircRNAs could be enriched by RNase R treatment (Wang et al., 2017a).

**FIGURE 5:**
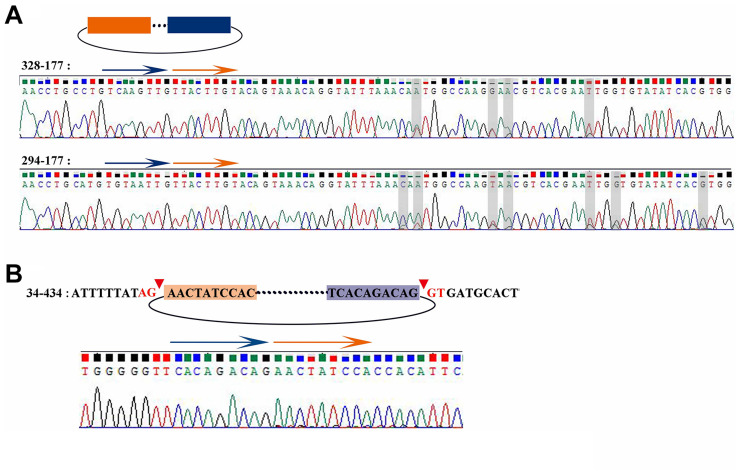
Sanger sequencing result of 328-177, 294-177, and 34-434. **(A)** Sanger sequencing of 328-177 and 294-177 confirmed the back-splicing junction in PCR products. Some overlapping peaks might mean that more than two transcripts formed by alternative splicing. **(B)** Sanger sequencing of 34-434 confirmed the back-splicing junction in PCR products.

According to the results of Sanger sequencing, three circRNAs, including 30–219, 21–352, and 28–273, showed the same sequences in the database, and the back-splicing junction was simultaneously found in their sequence (Figure 4D and Supplementary Figure 1A). The sequence identity of 328–177 and 294–177 was approximately 80.2%, and the sequencing results of the two RT-PCR products showed double peaks at specific positions, which suggested that back-splicing occurred on the exon where alternative splicing existed (Figure 5A). A similar situation was observed on 50–170 and 22–545 (Supplementary Figure 1B).

The sequence 34–434 also had the back-splicing junction, while it was 29 bases ahead of the predicted position. The two sites of back-splicing, which simultaneously followed the GT-AG rule, were found in the database and confirmed via Sanger sequencing (Figure 5B). That 34–434 could not be amplified by convergent PCR primers may be due to the primer designed on the 29 bases behind the back-splicing junction.

### Binding Prediction of CircRNAs and miRNAs on GSTP1

**FIGURE 6:**
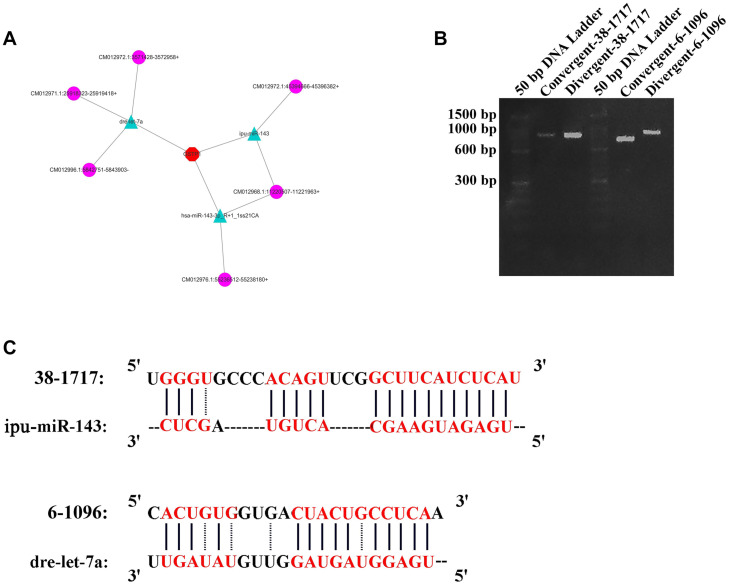
CircRNA analysis for GSTP1. **(A)** Network of mRNA, miRNAs, and circRNAs. **(B)** Amplification result of RT-PCR for 38-1717 and 6-1096. **(C)** Combination of circRNAs and miRNAs.

One crucial function of circRNAs is to act as a miRNA sponge to absorb miRNA and regulate gene expression. According to software prediction, the genes in the miRNAs database could be matched with the circRNA database to obtain the predicted correspondence. Based on a previous research on GSTP1 (Ge et al., 2017), a total of three miRNAs (dre-let-7a, ipu-miR-143, and hsa-miR-143-3p_R + 1_1ss21CA) that bind to the 3′UTR region of GSTP1 were identified in the liver tissue of *C. plagiosum*. Besides, these miRNAs were matched with circRNAs. According to the GSTP1 network of mRNA, miRNAs, and circRNAs (Figure 6A), six circRNAs were predicted to combine the three miRNAs. Among them, two circRNAs (named 38-1717 and 6-1096, Table 4) were analyzed and predicted to be able to bind miRNAs. The divergent and convergent PCR primers were designed to verify circRNAs, which showed that the two predicted circRNAs were both present in the shark liver tissue (Figure 6B). RNA hybrid software analysis indicated that the minimum free energy of the circRNAs and miRNAs was approximately –30.1 kcal/mol (38-1717 and miR-143) and –28.9 kcal/mol (6-1096 and dre-let-7a) (Figure 6C), indicating their binding strength and possible ability bind to miRNAs to regulate GSTP1 expression.

**TABLE 4:** Information on the GSTP1-circRNAs.

circRNA ID	Number of BSj reads	Predicted sequence length	circRNA name	Length of RT-PCR product	
				Convergent	Divergent
CM012972.1:45394666-45396382 +	38	1717	38-1717	867	897
CM012971.1:25918323-25919418 +	6	1096	6-1096	777	907

### Interaction Study of Identified CircRNAs With miRNA

Through the constructed recombinant vector and microRNA mimics, four recombinant miRNA groups: psiCHECK 2 + microRNA mimics-ipu-miR-143 empty plasmid control group, psiCHECK 2-38-1717 + microRNA mimics-ipu-miR-143 experimental group, psiCHECK 2 + microRNA mimics-dre-let-7a empty plasmid control group, psiCHECK 2-6-1096 + microRNA mimics-dre-let-7a were transfected into 293T cells, after 48 h incubation, the luciferase activity was evaluated by Dual Luciferase Reporter Gene Assay Kit (Yeasen, China).

**FIGURE 7:**
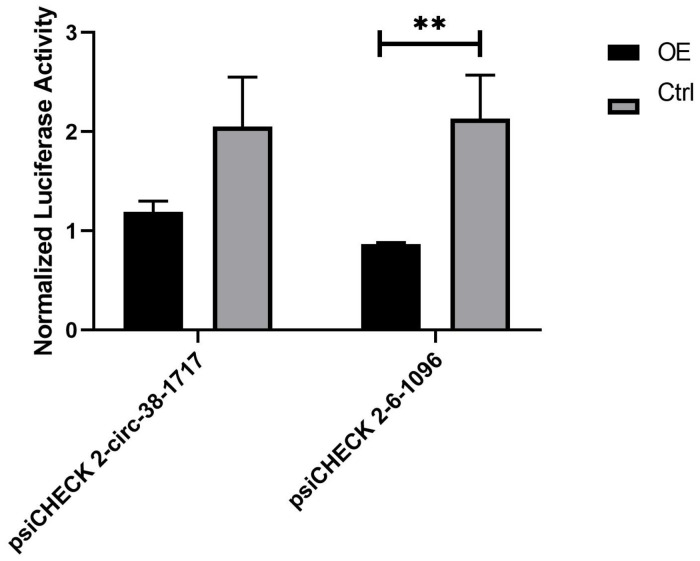
Diagram of the interaction between circRNA and miRNA. They are the psiCHECK 2-circ-38-1717-OE group and psiCHECK 2-circ-6-1096-OE group and its corresponding control group. The *t*-test analysis shows that psiCHECK 2-circ-6-1096-OE and its control group have significant difference. Statistical analysis was performed by Prism 8, ^∗∗^*P* ≤ 0.01, indicated significant difference.

During the analysis of the measured data, and 3 replicates were needed for the experimental group. The first is calculate the ratio (F/R) of Firefly Luciferase/Renilla Luciferase for each hole. Then calculate the average of the three duplicate wells of 38-1717-miRNA-Ctrl and 6-1096-miRNA-Ctrl, called average1. Then use the average1 of the 38-1717-miRNA-Ctrl and 6-1096-miRNA-Ctrl control groups as the standard 1, and use the psiCHECK 2-circ-38-1717-OE group and psiCHECK 2-circ-6-1096-OE F/R three repeated values (Divide by average1 to get three ratios (equivalent to a normalization). Finally, the average value of the three repeated wells of 38-1717-miRNA-Ctrl and 6-1096-miRNA-Ctrl and the three of psiCHECK 2-circ-38-1717-OE group and psiCHECK 2-circ-6-1096-OE. The ratio value is input to Graphpad prism8.0.1 to draw the mutual mapping of circRNA and miRNA (Figure 7). The data showed that the experimental group has a large degree of fluorescence value reduction, indicating that circRNA and miRNA interact, suggesting that the prediction is correct, and the two groups of experimental groups and control groups are analyzed by *t*-test (^∗^indicated significant difference).

## Discussion

In recent years, the roles of circRNAs in tumor immunity regulation and immunotherapy have been demonstrated by an increasing number of studies focusing on their function as an miRNA sponge (Xu et al., 2018). CircRNA is particularly less explored in marine animals. *C. plagiosum* is a demersal cartilage fish, with no relative circRNA identified and characterized currently. In this research, 4,558 unique circRNAs were systematically identified, among which more than 45% had a sequence size of more than 10,000 bp. In all 4,558 circRNAs, 967 circRNAs (approximately 20%) contain more than four BSj reads, in which more than 45% are longer than 10,000 bp. This phenomenon is uncommon relative to other species (Pallavicini et al., 2013b; Maass et al., 2017) because none of current data of other fish showed a high percentage of long circRNAs (Pallavicini et al., 2013a). We speculate that this phenomenon might be due to the incomplete genomic information on *C. plagiosum* or the presence of long circRNAs, which could absorb more miRNAs and thus might have regulatory effect on multiple pathways.

Eight randomly selected shark circRNAs were verified, and findings showed that circRNA splicing follows the GT-AG rule. Sequencing results indicated that back-splicing could occur on the exon where alternative splicing existed, which means that alternative splicing occurs not only in mRNA transcription but also in back-splicing. During evolution, sharks might have evolved complex RNA splicing processes to meet their various physiological needs.

GSTs are primarily involved in cellular defense against toxic compounds in most living organisms, and they play an important role in immune responses. Mapping the mRNA–miRNA–circRNA network relationship for GSTP1 may provide information in the further study GSTP1 function in sharks. A complete mRNA–miRNA–circRNA network could not be established because the transcriptome information on *C. plagiosum* is incomplete. Nevertheless, the construction and analysis of the relationship network could be established according to the target gene searching sequence, which would be valuable for subsequent studies on other genes. The relationship between miR-dre-let-7a and circRNA_-6-1096 was confirmed in a dual-luciferase reporter assay. It confirms the authenticity of our prediction and provides a basis for us to verify the interation of CircRNA as a miRNA sponge, and also provides a promising target for various liver diseases in the future.

## Conclusion

In this study, a total of 4,558 circRNAs were analyzed and identified in the liver tissue of *C. plagiosum*. At least one back-splicing junction reads (BSj reads) was obtained in the identified circRNAs, and further analysis revealed that *C. plagiosum* circRNAs were not evenly distributed on the chromosomes but followed the GT-AG rule during cyclization. Alternative back-splicing existed in shark circRNAs. We found three miRNAs (dre-let-7a, ipu-miR-143, and hsa-miR-143-3p_R + 1_1ss21CA) binding to the 3′UTR region of GSTP1 and generated a potential mRNA–miRNA–circRNA network for GSTP1. The relationship between miR-dre-let-7a and circRNA_-6-1096 was confirmed in a dual-luciferase reporter assay. Confirmed our prediction and confirmed the biological function of circRNA as a sponge of miRNAOur findings may contribute to existing studies on *C. plagiosum* circRNAs in the cartilaginous fish and provide a basis for characterizing the functions of circRNAs in marine animals with a focus on the circRNAs in the liver tissue of *C. plagiosum* (Meng et al., 2018).

## Data Availability Statement

The circRNA sequencing data is available in NCBI at https://www.ncbi.nlm.nih.gov/geo/query/acc.cgi?acc=GSE157160.

## Ethics Statement

The animal study was reviewed and approved by the Institutional Animal Care and Use Committee of the Zhejiang Sci-Tech University.

## Author Contributions

WZ: investigation and writing of the original draft. PQ: investigation, writing of the original draft, and editing. XG: writing, review and editing, and use of software. LH: formal analysis and validation. CW: writing and review and editing. GC and JC: validation, project administration, and revision of the manuscript. LW: conceptualization, methodology, and revision of the manuscript. ZL: conceptualization and resources. All authors read and approved the final manuscript.

## Conflict of Interest

LH was employed by company Hangzhou Hongqiao Sino-Science Gene Technology Co., Ltd. The remaining authors declare that the research was conducted in the absence of any commercial or financial relationships that could be construed as a potential conflict of interest.

## Footnotes

1 http://www.circrna.org
