# Supplementary material for: Identification of circRNAs in the Liver of Whitespotted Bamboo Shark (Chiloscyllium plagiosum)
Source: Front Genet. 2020 Dec 11;11:596308. doi: 10.3389/fgene.2020.596308 (PMC7759564; doi:10.3389/fgene.2020.596308)
Supplement: Supplementary file 1 [file Presentation_1.pdf]

Supplementary Figures

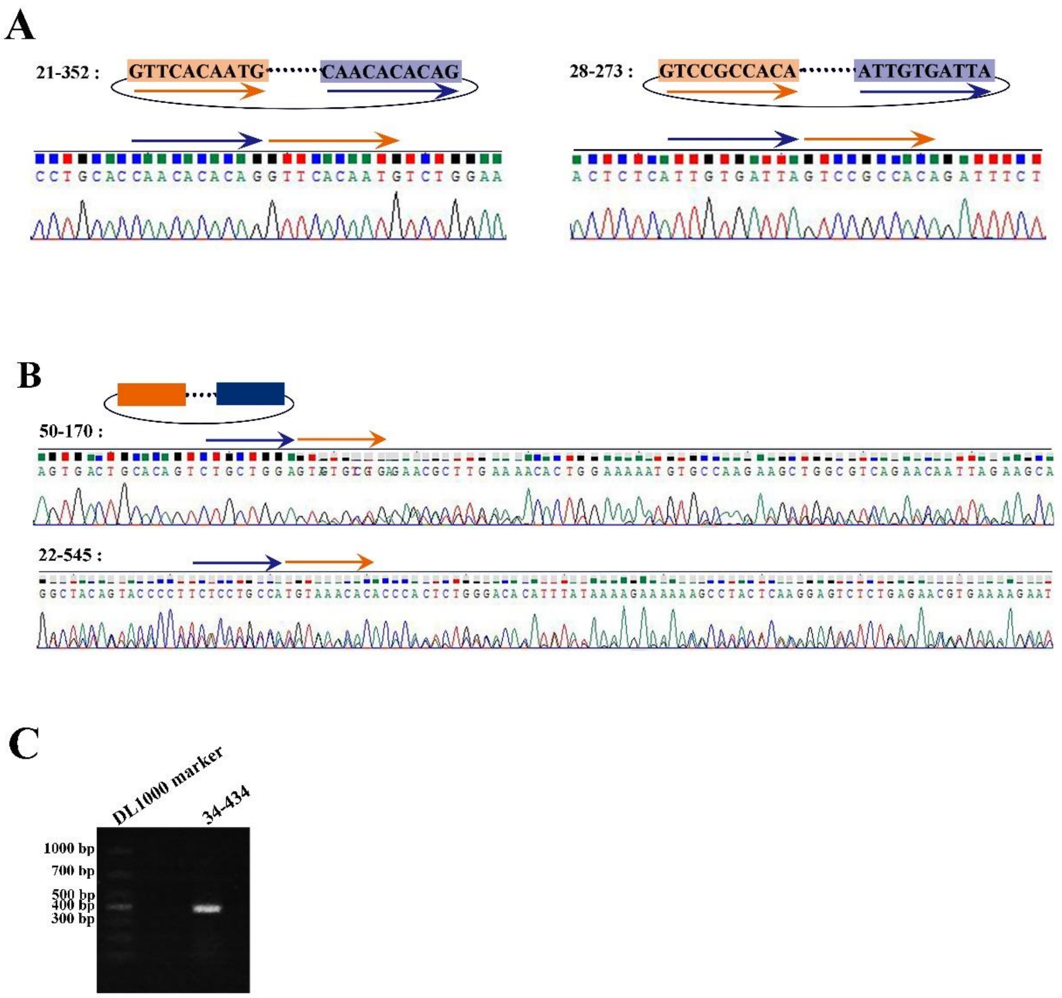

Supplementary Figure 1. Supplementary figure. (**A**)Sanger sequencing of 21-352 and 28-273 confirming the back-splicing junction in PCR products. (**B**) Sanger sequencing result of 50-170 and 22-545. (**C**) Amplification result of 34-434 using divergent PCR primers
